# The Evidence for the Spread and Seeding Capacities of the Mutant Huntingtin Protein in *in Vitro* Systems and Their Therapeutic Implications

**DOI:** 10.3389/fnins.2017.00647

**Published:** 2017-11-28

**Authors:** Maria Masnata, Francesca Cicchetti

**Affiliations:** ^1^Axe Neurosciences, Centre de Recherche du CHU de Québec-Université Laval, Quebec, QC, Canada; ^2^Département de Psychiatrie & Neurosciences, Université Laval, Quebec, QC, Canada

**Keywords:** Huntington's disease, Parkinson's disease, Alzheimer's disease, prions, tau, β-amyloid, α-synuclein, cell culture

## Abstract

Neurodegenerative disorders are not only characterized by specific patterns of cell loss but the presence and accumulation of various pathological proteins—both of which correlate with disease evolution. There is now mounting evidence to suggest that these pathological proteins present with toxic, at times prion-like, properties and can therefore seed pathology in neighboring as well remotely connected healthy neurons as they spread across the brain. What is less clear, at this stage, is how much this actually contributes to, and drives, the core pathogenic events. In this review, we present a comprehensive, up-to-date summary of the reported *in vitro* studies that support the spreading and seeding capacities of pathological proteins, with an emphasis on mutant huntingtin protein in the context of Huntington's disease, although *in vivo* work remains to be performed to validate this theory in this particular disease. We have further reviewed these findings in light of their potential implications for the development of novel therapeutic approaches.

## Are “prionic” proteins pathogenic and found in all neurodegenerative diseases?

The discovery of Lewy-body pathology within fetal ventral mesencephalic cells grafted in patients with Parkinson's disease (PD) years earlier has radically changed our views on the potential pathogenic mechanisms underlying sporadic neurodegenerative disorders of the central nervous system. This observation, initially reported by two independent teams (Kordower et al., 2008; Li et al., 2008), has led to the theory that the pathogenic α-synuclein protein can spread from the diseased brain to healthy tissue and cause protein aggregation and cellular dysfunction in a prion-like fashion (Olanow and Prusiner, 2009; Brundin et al., 2010; Soto, 2012). Indeed, it has been demonstrated both *in vitro* and *in vivo* that α-synuclein, the main component of Lewy bodies, can be released into the extracellular space and then be internalized by neighboring neurons (Desplats et al., 2009; Hansen et al., 2011), acting as a toxic agent that could seed pathology in the process. Furthermore, intracerebral inoculation of brain homogenates derived from aged α-synuclein transgenic mice, or injections of synthetic α-synuclein preformed fibrils, accelerates the formation of protein aggregates and precipitates neurological dysfunction in small animals (Luk et al., 2012a,b). It is also now known that there is pathology remote from the injection sites in these types of studies, which further supports an intercellular transneuronal spread of protein, as has also been demonstrated in rodent allografts placed in animals expressing human α-synuclein (Angot et al., 2012). In the latter study, human α-synuclein was shown to co-localize with markers of endosomes and exosomes (Angot et al., 2012), which could represent one of the routes by which the protein is transferred (Goedert et al., 2010; Angot et al., 2012). This mode of protein spread and disease propagation has been shown experimentally with several other proteins including amyloid, tau, SOD1, TDP-43 and FUS (Soto, 2012; Jucker and Walker, 2013; Guo and Lee, 2014). However, these studies have proposed a number of other putative mechanisms for protein spread which include nanotubes (Costanzo et al., 2013; Abounit et al., 2016a,b), vesicular transport (Angot et al., 2012; Lee et al., 2012), endocytosis (Hansen et al., 2011; Wu et al., 2013; Ruiz-Arlandis et al., 2016) or even direct penetration of the plasma membrane (Ren et al., 2009).

What is emerging from all this work is that a number of these processes may be common to all neurodegenerative disorders, not just sporadic but also monogenic diseases such as Huntington's disease (HD) (Brundin et al., 2010; Soto, 2012; Cicchetti et al., 2014). In HD, recent evidence has added weight to the idea that the mutant huntingtin protein (mHTT)—the genetic product that defines the disease—can propagate from cell-to-cell. *In vivo* observations for the ability of mHTT to travel across synapses has been collected in embryonic human stem cells differentiated into neurons and implanted in R6/2 mice, a transgenic mouse model of HD (Pecho-Vrieseling et al., 2014). It has also been demonstrated by expressing the human HTT gene (138 CAG) within olfactory receptor neurons which was subsequently found in the synaptically connected large posterior neurons in the brain of drosophila (Babcock and Ganetzky, 2015). Various cell types (HEK), including neuron-like cells (Cos-7 and PC-12), have been shown to be capable of internalizing synthetic mHTT aggregates from the extracellular milieu (Yang et al., 2002; Ren et al., 2009). The aggregates can be either translocated to the nucleus where their pathogenic effects on transcription can be exerted leading to cell dysfunction and death (Yang et al., 2002) or act as seeds for further protein aggregation within the cell itself (Herrera et al., 2011). Prion-like spread has also been suggested to take place following the phagocytosis of mHTT aggregates by glial cells in a *Drosophila* model of HD. The engulfed aggregates gained access to the cytoplasm of microglia where they interacted with soluble huntigtin (HTT), initiating a prion-like dissemination of pathology (Pearce et al., 2015). Although there are no other reports on the role of immune cells in mHTT propagation (Weiss et al., 2012), this is a likely scenario that may, at least partly, contribute to HD pathology and that certainly cannot be dismissed at this stage.

The notion that neurodegenerative disorders, including HD, share several features with classical prion diseases is gaining momentum—although this concept remains more debated for this disease and more *in vivo* work is clearly needed to substantiate the initial *in vitro* claims. Notwithstanding, at least two fundamental questions remain to be answered: What are the routes by which the pathological proteins, such as mHTT, propagate and therefore spread and seed their pathogenic effects? And can any of these routes be blocked to prevent disease dissemination? The aim of this manuscript is to review the *in vitro* evidence for the spreading and seeding capacities of mHTT and what implications this has for the development of novel therapeutic approaches.

## Is mHTT equally toxic to striatal and cortical neurons?

One of the most striking pathological features of HD is the massive loss of striatal projection neurons. However, the degeneration is not confined to this structure and cortical areas as well as a number of other sites all show cell loss early on in the disease process. However, cells in both of the cortical and striatal areas are tightly connected synaptically. They both express mHTT but their vulnerability to early degeneration in HD remains elusive, as does the chronology in which they degenerate with respect to one another. *In vitro* models in which mHTT expression is induced by high capacity adenoviral (Dong et al., 2012) or lentiviral vectors (Zala et al., 2005), have revealed that cortical neurons accumulate a significant number of inclusion bodies, but with no clear toxic consequences. In contrast, striatal neurons develop major morphological changes that are accompanied by the loss of neurofilaments and ultimately cell death, despite the fact that mHTT aggregates are rarely seen within these cells (Zala et al., 2005; Dong et al., 2012). The reasons for this differential vulnerability have been further investigated in the BACHD mouse model by genetically inhibiting mHTT production in the striatum and cortex simultaneously, or in either structure alone. Inhibition of mHTT synthesis in both the striatum and cortex combined had the most significant beneficial impact on motor and psychiatric HD-related behavioral phenotypes (Wang N. et al., 2014) with an abrogation of striatal degeneration. This may imply that the content of mHTT within cortical cells changes the cell properties; i.e. by producing more glutamate which creates excitotoxicity within their target structures such as the striatum. However, it may also indicate that preventing the propagation of mHTT via the cortico-striatal circuit can have a considerable impact on the disease.

## Transneuronal/transynaptic propagation: how neuronal circuits enable mHTT spread

**Figure 1:**
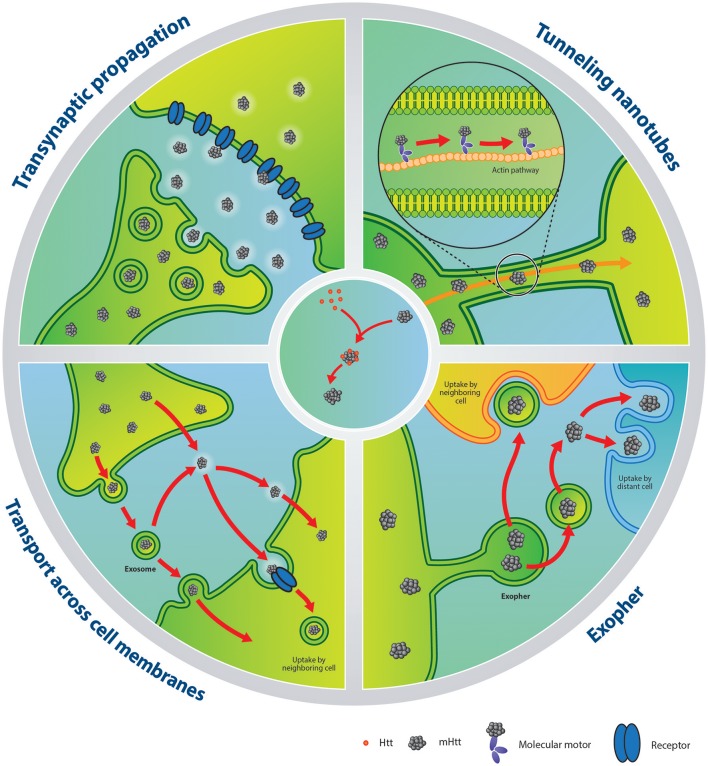
Putative mechanisms of mHTT spreading and seeding capacities**. Left upper panel**: illustration of transynaptic propagation of mHTT. **Right upper panel**: transport mechanism of mHTT via tunneling nanotubes. **Left lower panel**: mHTT can be released within exosomes or in a free form. After extrusion, exosomes carrying mHTT can fuse with the plasma membrane of a neighboring cell. Alternatively, mHTT can escape from the exosomal-vesicle into the extracellular compartment, with the same fate as the counterpart released directly as a free form. Finally, mHTT can be internalized by a recipient cell via receptor-mediated endocytosis or directly penetrate the plasma membrane. **Right lower panel**: In neurons of C. elegans, mHTT has been shown to be contained within exophers, an entity which resembles mammalian exosomes. Released exophers may be incorporated by adjacent or distant cells or secrete their contents into the milieu. **Central panel**: schematic of the seeding process of mHTT. The misfolded protein recruits HTT in an elongation process creating toxic aggregates. HTT, huntingtin; mHTT, mutant huntingtin.

The validity of the transynaptic propagation theory (Figure 1) has gained additional support with the work of Pecho-Vrieseling et al. (2014). In this study, three distinct models were used to investigate this concept. In one set of experiments, the authors generated mixed-genotype (R6/2-wild-type) cortico-striatal cultures, more specifically combining R6/2 striatal neurons with wild-type cortical neurons or wild-type striatal neurons with R6/2 cortical neurons. Functional R6/2 cortical-wild-type striatal networks were created and used to study long-distance mHTT propagation from the cortex to wild-type DARPP-32+ medium spiny neurons. However, this was not seen in the R6/2 striatum-wild-type cortical circuit as no significant amounts of mHTT deposits were detected in the wild-type cortex.

In a second set of experiments, the authors pursued the transynaptic propagation theory using embryonic human stem cells differentiated into neurons and tagged with GFP (hGFP) which they then transplanted into organotypic brain slices derived from R6/2 mice. Cell inoculations were performed within the cortex and striatum and the identity of the transplanted cells was confirmed by immunostainings of mature and structure-specific cellular markers such as Tbr1 (cortex) and DARPP-32 (striatum). Two major observations were made relating to the propagation of mHTT: from endogenous neurons of the R6/2 mice or to the freshly transplanted human cells. Firstly, two waves of mHTT accumulation were seen between the mouse neurons: one wave occurred at 3–4 weeks and a second took place between 6 to 8 weeks following the initiation of cultures. In the human transplanted cells, mHTT accumulation progressively increased within the striatum between 4 and 8 weeks at which point it plateaued, while in the cortex, the pattern of propagation was identical to that seen in mouse neurons. The impact of mHTT propagation was seen primarily on neurites, which became atrophied. This was accompanied by a concomitant loss of DARPP-32+ neurons, one of the main features of HD pathology. Finally, the location of mHTT aggregates was first identified in the cytoplasm and subsequently in the cell nucleus. It should be noted that the authors repeated the experiment using human pluripotent stem cells differentiated into neurons and also showed the transynaptic spread of mHTT from R6/2 host tissue to human grafts, indicating that this was common to different cell types.

A final set of experiments was performed *in vivo* where wild-type mice were injected with Q72-HTT-Exon1 and synaptophysin-GFP viral vectors into cerebral cortical layers. In this case, mHTT aggregates were detected predominantly in medium spiny neurons expressing GFP which were innervated by cortical projection neurons previously transduced with the viruses, suggesting an active role for cortical projections in mHTT propagation to striatal neurons. Post-mortem analyses of grafted hGFP-neurons in cortical areas of 4-week-old R6/2 mice further confirmed these observations.

The seminal work of Pecho-Vrieseling et al. (2014) confirmed the hypothesis brought forward by Cicchetti et al. (2014) that mHTT could propagate transynaptically between disease and healthy tissue. But exactly how mHTT is transported whithin neurons remains unanswered. To understand this, microfluidic culture devices have been employed in which neuronal somata could be isolated from their processes and other cell types. With this system, it was shown that synthetic α-synuclein fibrils can be transported both anterogradely and retrogradely (Volpicelli-Daley et al., 2011; Freundt et al., 2012; Brahic et al., 2016), as well as to be released and taken up by second order neurons (Freundt et al., 2012). Similar observations have been made with Aβ42 (Freundt et al., 2012; Brahic et al., 2016), tau (Wu et al., 2013; Brahic et al., 2016) and HTTExon1 fibrils (Brahic et al., 2016). However, HTTExon1 fibrils showed limited anterograde transport, although retrograde transport efficiency was similar to that seen with α-synuclein fibrils (Brahic et al., 2016; Table 1).

**Table 1:** *In vitro* evidence of mHTT spreading capacities.

**Mechanism**	**Protein form**	**Cell model**	**Observations**	**References**
Transynaptic propagation	Endogenous mHTT from R6/2 mice	*Ex vivo* mixed cortico-striatal cultures from R6/2 or wild type mice	Propagation of mHTT from R6/2 cortical to wild-type striatal neurons Significant vulnerability of striatal neurons in comparison to cortical neurons	Pecho-Vrieseling et al., 2014
	Endogenous mHTT from R6/2 mice	Human ESCs and human iPSC differentiated into neurons transplanted into organotypic brain slices of R6/2 mice	Propagation of endogenous mHTT from murine host tissue to grafted hGFP neurons followed by progressive neurodegeneration of recipient hGFP neurons	
TNTs	Transfection with GFP-480-68Q (donor); mCherry (acceptor)	Co-culture of CAD transfected cells (68Q or mCherry) Co-culture of transfected primary CGNs (68Q or mCherry)	Transfer of GFP-480-68Q to both CAD and CGN neuronal cells via TNTs	Costanzo et al., 2013
Vesicular transport				
*Exosome*	HD143F-derived exosomes	Co-culture of HD143F and NSCs	Spread of mHTT from HD143F to NSCs	Jeon et al., 2016
		NSCs exposed to HD143F-derived exosomes	Spread of exosomes-containing mHTT in NSCs	
*Exopher*	Genetically-engineered expression of Q128	*C. elegans*	Q128 gene expression increases the production of exophers Exopher content, including organelles, protein and mHTT, is found in remote cells of the *C. elegans*	Melentijevic et al., 2017
Endocytosis	Fibrillar Alexa488-HTTExon1Q44 and/or polyQ44	Undifferentiated and differentiated mouse and human neuroblastoma cells (N2A and SH-SY5Y)	Internalization and intracellular localization of HTTExon1Q44 and PQ44 fibrils in both types of neuroblastoma cells Fibrillar HTTExon1Q44 uptake via clathrin-dependent endocytosis No mechanisms evaluated for PQ44 fibrils	Ruiz-Arlandis et al., 2016
Direct penetration of plasma membrane	Synthetic K2Q44K2 fibrils	HEK; HeLa; Cos-7; CHO; N2A	Breach plasma membranes by K2Q44K2 fibrils in all cell types tested	Ren et al., 2009
	Transfection with ChFP-HTTQ25 and synthetic K2Q44K2 fibrils	HEK	Recruitment of soluble HTT forms into IBs by synthetic K2Q44K2 fibrils in transfected HEK cells	
	Chemically synthesized Q42, NLS-Q42 and NLS-Q20 fibrils	Cos-7; PC-12	In the nuclei, smaller aggregates are more toxic than larger ones in both cell types tested	Yang et al., 2002
Unknown	Transfection with 25/103QHTT-V1 and 25/103QHTT-V2	Co-culture of H4 cells expressing 103QHTT-V1 and HEK cells expressing 103QHTT-V2	Polymerization and cell-to-cell transmission of HTT oligomers	Herrera et al., 2011
	Exposure to conditioned medium derived from GFP-mHTT-Q19 or GFP-mHTT-Q103 transfected HEK cells	SH-SY5Y cells	Presence of exogenous mHTT protein (Q19 and Q103) within recipient SH-SY5Y cells	Jeon et al., 2016

*CAD, mouse catecholaminergic neuronal cell line; CGNs, cerebellar granule neurons; CHO, epithelial-like cell line from Chinese hamster ovary; Cos-7, fibroblast like cell lines derived from monkey kidney; ESCs, embryotic stem cells; H4, human brain neuroglioma cells; GFP, green fluorescent protein; HD, Huntington's disease; HD143F, human fibroblast derived from Huntington's disease patient carrying 143 polyglutamine repeats; HEK, human embryonic kidney cells; HeLa, human uterine cervical carcinoma cells; hGFP neurons, human GFP positive neurons; HTT, huntingtin; IBs, inclusion bodies; iPSC, induced pluripotent stem cells; mHTT, mutant huntingtin; NSCs, neural stem cells; N2A, mouse neuroblastoma cell line; NLS, nuclear localization signals; PC-12, cell line from pheomochromocytoma of the rat adrenal medulla; PolyQ, polyglutamine; SH-SY5Y, human neuroblastoma cell line; TNTs, Tunneling nanotubes; V1, Venus protein half; V2, Venus protein half 2*.

It has now been demonstrated, *in vitro, in vivo* and in post-mortem human samples, that mHTT is able to disrupt vesicular and mitochondrial trafficking by recruiting and sequestering key elements of the axonal trafficking machinery, such as normal HTT, within aggregates (Trushina et al., 2004). This could explain the differences observed in the axonal transport between mHTT and other types of protein fibrils, in particular the fact that, after retrograde transport, HTTExon1 fibrils accumulate intracellularly, while more than half of the α-synuclein and Aβ42 fibrils are released into the media (Brahic et al., 2016). It is clear that the properties of mHTT do not simply allow for it to be transferred between cells but that it is also capable of triggering aggregate formation using endogenous proteins within the recipient cell - and by so doing display prionic characteristics.

## Tunneling nanotubes: a channel for disease dissemination

Tunneling nanotubes (TNTs) are small entities that serve as a communication bridge between cells (Abounit and Zurzolo, 2012). Using adhesion proteins and fusion molecules (SNARE or viral fusion proteins), TNTs can change their configuration to merge to other cellular surfaces (Marzo et al., 2012). Neurons and various other cell types have the ability to produce these temporary and retractable protrusions which are made of F-actin strains and lipid bilayers containing organelles, plasma membrane components and ions such Ca^2+^ which are key to cell signaling (Smith et al., 2011). In normal physiological conditions, they have been noted to participate in cell development (Gurke et al., 2008), contribute to immune responses (Watkins and Salter, 2005), engage in regeneration processes (Wang et al., 2011) as well as facilitating electrical conduction between cells (Smith et al., 2011).

Within the F-actin path forming the TNTs, molecular motors can be hijacked to transport pathogens and prion-like proteins (Gousset et al., 2009; Abounit and Zurzolo, 2012; Figure 1). This has been demonstrated in mouse catecholaminergic neuronal cells for α-synuclein (Abounit et al., 2016a) and in both mouse catecholaminergic neuronal cells and mouse cerebellar granule neurons for mHTT (Costanzo et al., 2013). Furthermore, HTT and mHTT have been shown to form strong interactions with phospholipid bilayers suggesting that they can drift on F-actin streams and lipid surfaces (Marzo et al., 2012). More specifically, α-synuclein fibrils taken up from the media are almost exclusively found embedded in endolysosomal vesicles (Abounit et al., 2016a) within TNTs. In contrast, mHTT fibrils are identified in a free form state within the cell cytoplasm (Ren et al., 2009; Table 1) and their colocalization with vimentin hints to potential transport within aggresome-like structures (Costanzo et al., 2013). Although TNT formation can offer a defense mechanism for expelling material that the cell cannot digest/degrade—for example fibrillar amyloids—this system is not sufficient to completely restore the cell's health. Additionally, cells that contain pathological proteins produce more TNTs (Costanzo et al., 2013; Abounit et al., 2016a,b), creating opportunities for pathological protein transfer and potentially facilitating the seeding process in naïve cells.

## Exosomes and exophers as mHTT cargo carriers

It has now been clearly shown that misfolded proteins can be found within extracellular vesicles and that they can be carried and delivered to a recipient cell using this means (Figure 1). The demonstration that this applies to mHTT as well has also been shown using exosomes extracted from fibroblasts derived from a severe juvenile HD case harboring 143 CAG repeats (HD143F) and which were exposed to differentiated neuronal stem cells derived from embryonic cortical mouse tissue. After 4 days of contact between the exosomes and the cultured cells, the internalization of mHTT was detectable. Similar results were achieved when HD143F-derived exosomes were incubated with SH-SY5Y cells, confirming the spreading capacity of mHTT via exosomes in various cell types (Jeon et al., 2016; Table 1). Most strikingly, HD143F exosomes injected intraventricullarly into new-born wild-type animals led to the development of motor and cognitive HD-related behavioral phenotypes (Jeon et al., 2016). This study provided evidence that exosomes carrying mHTT could participate in disease induction/manifestation both *in vitro* and *in vivo*.

More recently, the *C. elegans* model has served to identify a novel vesicular entity capable of incorporating and extruding dysfunctional organelles as well as protein aggregates. This transporter was named an “exopher” (Melentijevic et al., 2017). Despite their larger size and the fact that they are born from a different genesis process, exophers resemble, in many ways, exosomes found in mammalian cells. They are released in a multiple-step process which includes protrusion, elongation and separation (Figure 1), emerging from the soma of different neurons, independently of cell division or cell death (Melentijevic et al., 2017). Their production is triggered by cellular stress, disruption of physiological conditions or, importantly, by the presence of inclusion bodies such as mHTT aggregates (Melentijevic et al., 2017). For example, the expression of aggregable HTT-Q128 in the genome of the nematode causes impairments of the normal functions of chaperones, autophagy pathways, ubiquitin-proteasome systems and provokes the collapse of the integrity maintenance machinery in cellular elements. The accumulated stress increases the production of exophers in touch-responsive ALMR neurons expressing Q128 in this animal which in fact serves as defense mechanisms to restore the cell's equilibrium (Melentijevic et al., 2017). However, the downside of exopher production is that they may, like exosomes, enable cell-to-cell delivery of insoluble pathogenic proteins (Melentijevic et al., 2017; Table 1).

## Accessing the membrane of neighboring cells by endocytosis

Endocytosis is a cellular mechanism that controls various cellular functions such as internalization and recycling of plasma membrane components/ligands as well as the uptake and degradation of macromolecules and extracellular particles. The process begins with modifications of the plasma membrane, the generation of endocytic vesicles, which then matures into *early* and *late* endosomes, and finally leads to the degradation of the vesicle content through fusion with lysosomes (Miaczynska and Stenmark, 2008).

There is now compelling evidence that proteins, such as α-synuclein, can be incorporated into cells via clathrin-dependent endocytosis (Oh et al., 2016; Figure 1). Using undifferentiated and differentiated N2A cells exposed to HTTExon1Q44 and treated with various pharmacological compounds, clathrin-dependent endocytosis was identified to participate in mHTT uptake. In this particular study, HTTExon1Q44 fibrils were found in early endosomes of undifferentiated cells. This was observed as early as 6 h post-exposure to the toxic material or in both early endosomes and lysosomes 24 h post-exposure. At 48 h, concentrations of fibrils were unchanged within the lysosomes, but were found at lower concentrations in the early endosomes (Ruiz-Arlandis et al., 2016; Table 1). It was speculated that mHTT concentrations inside endosomes was lower because mHTT had been delivered to the lysosomes. In contrast, the stable concentrations of mHTT inside the lysosomes may have resulted from an equilibrium between the quantities of ingested vs. degraded fibrils. It is also feasible, according to the authors, that this observation reflects the saturation of the lysosome degradation machinery. It has been noted that the fate of the same fibrils differed in differentiated N2A, where co-localization within early endosomes could not be firmly established. Rather, mHTT fibrils were present within lysosomes, perhaps testifying to the cell's attempt to eliminate toxic mHTT through lysosomal degradation.

The idea that the turnover of HTT and mHTT aggregates is dependent on the endosomal–lysosomal system finds supports in another study in which it has been reported that following transfection, free HTT accumulated in the cytoplasm and inside autophagosome-like vacuoles, while mHTT localized both to the cytoplasm and nucleus. The presence of mHTT in vacuoles modified the morphology of the cells, which appeared atrophied, further implying that endosomal-lysosomal-vacuolar pathway activation may be responsible for this type of cell death (Kegel et al., 2000). HTT and mHTT have also be shown to co-localize with late endosome and lysosome markers and this has led to speculation that this was due to the lysosome's capacity to mediate the secretion of proteins fusing with the plasma membrane through an active calcium dependent process (Figure 1). Endosome ablation, calcium chelator or silencing synaptotagmin 7 (lysosome-specific calcium sensor) inhibited mHTT secretion, providing evidence for the involvement of late endosomal/lysosome pathway in secreting mHTT and, in smaller amounts, wild-type HTT (Trajkovic et al., 2017).

## mHTT transmission by direct penetration of plasma membranes

Release and uptake are the basic elements of a prion-like cell-to-cell protein propagation process. In order to dissect the mechanisms of transit between cells, artificially manufactured polyQ proteins were incorporated to the media of different mammalian cell cultures to verify and monitor their potential internalization, location after uptake and consequent toxic effects. Internalization of liposome-coated (4 h post-infection) and uncoated fibrils (24–48 h post-infection), characterized by 42 CAG repeats, were detected in both Cos-7 and PC-12 cells (Table 1). In this context, the presence of synthetic aggregates within the cytoplasm did not interfere with the physiological functions of the acceptor cells. To mimic the physiological expression of aggregates in the nucleus (Saudou et al., 1998)—which has been suggested to induce greater toxicity—the penetrance of the nuclear membrane was facilitated by the modification of the polyQ peptides by adding nuclear localization signals (NLS). While the F-Q42 was detected in proximity to the nuclear membrane, F-NLS-Q42 was easily delivered to the nucleus. Cell death was frequent in Cos-7 and PC-12 cells administered with F-LNS-Q42 or F-LNS-Q20 and was strictly associated with smaller sized fibrils (Yang et al., 2002), suggesting that the presence of polyQ into the nucleus is associated with high toxicity, independent of the polyQ length (Table 1).

A more elaborate study conducted in a wide variety of mammalian cells (Cos-7, HEK, N2A, CHO, and HeLa) revealed that only 1 h after contact, synthetic K2Q44K2 peptides were found within the cytoplasm, co-localizing with cytosolic quality control components in all cell types. Although their location was expected within the endosomal compartment (Lee et al., 2008), no co-localization with endosome, lysosome and autophagosome markers was revealed suggesting that fibrils were found free within the cytoplasm and were thus available to aggregate with other proteins including HTT. Transmission electron microscopy further uncovered that K2Q44K2 could reach the intracellular compartment by physically breaching plasma membranes (Figure 1; Table 1).

The hypothesis of free Q44 fibrils interacting with HTT was more specifically investigated by assessing the aggregation state of cyan fluorescent protein (CFP)-tagged HTTExon 1 Q25 (CFP–HTTQ25) in HEK cells after exposure with K2Q44K2. CFP fluorescence in cells not yet exposed to polyQ fibrils showed a diffuse nucleocytoplasmic distribution, as expected of the soluble HTTQ25 fragment. Following incubation with K2Q44K2 fibrils, CFP florescence co-localized with the fibrils in distinct puncta, suggesting the recruitment of the Q25 soluble fraction into the aggregates. Using other forms of fibrils (non-fibrillar HTTQ18 and fibrillar HTTQ51) in the same cell culture conditions, cytosolic nucleation was induced by fibrillar polyQ peptides (Ren et al., 2009; Figure 1; Table 2). This technique was used in several studies providing evidence for mHTT seeding capacities in different cell lines such as HeLa (Trevino et al., 2012) or human and murine neuroblastoma cells (Ruiz-Arlandis et al., 2016). Additionally, exposure of PC-12—that can inducibly express truncated exon1—with cerebrospinal fluid derived from postmortem samples of HD patients or living BACHD transgenic rats, showed that mHTT could trigger the aggregation process (Tan et al., 2015; Figure 1; Table 2).

**Table 2:** *In vitro* evidence of mHTT seeding capacities.

**Protein form**	**Cell model**	**Observations**	**References**
Co-transfection with poly25Q EGFP and poly104Q c-Myc	Cos-1	Co-aggregation of normal length and extended polyQ tracts into cell cytoplasm	Kazantsev et al., 1999
Co-transfection with poly25Q nucleolin EGFP and poly104Q c-Myc	Cos-1	Heterogeneous aggregates of 104Q c-Myc and 25Q-nucleolin- EGFP within cell nuclei Appearance of homogenous cytoplasmic aggregates with the expression of 104Q c-Myc only	
Transfection with 104Q nucleolin EGFP and 104Q c-Myc	Cos-1	Extended polyglutamine colocalization in both the cytoplasm and nucleus with the coexpression of 104Q nucleolin EGFP and 104Q c-Myc Interactions between polyglutamines causes relocation 104Q c-Myc into the nucleus	
Co-transfection of HA-HDQ20 and/or HA-HDQ32 with GFP-HDQ72	Cos-1	Elongated HTT polyQ fragments can recruit wild-type HTT	Busch et al., 2003
Transfection with CFP-Q25HTTExon1 and exposure to K2Q44K2 fibrils	HEK	Co-localization of CFP-Q25HTTExon1 with K2Q44K2 induces amyloid nucleation in a sequence-specific manner	Ren et al., 2009
Transfection with ChFP-HTTExon1Q25 and exposure to positive, neutral or negatively charged FITC-labeled Q44 fibrils	HEK	Induction of nucleation within the cytoplasm of ChFP- HTTExon1Q25 transfected cells by all three types of fibrils (i.e., positive, neutral, and negative net charges)	Trevino et al., 2012
Transfection with ChFP-HTTExon1Q25 and exposure to non-fibrillar K2Q44K2 aggregates	HEK	Reduced internalization of non-fibrillar K2Q44K2 and nucleation of cytoplasmic ChFP-HTTExon1Q25 in comparison to fibrillar forms	
Transfection with ChFP-HTTExon1Q25 and exposure to HTTExon1Q44 or Q44 fibrils	HeLa	Internalization of HTTExon1Q44 and Q44 fibrils into HeLa cells and nucleation within the cytoplasm ChFP-HTTExon1Q25 transfected cells HTTExon1Q44 fibril internalization is less efficient than for the Q44 fibrils	
Transfection with HTT(Q25)CFP/YFP and exposure to HTT Q50 fibrils	C17.2	Heparan sulfate proteoglycans-independent internalization of Q50 fibrils and nucleation with exogenous HTT(Q25)CFP/YFP	Holmes et al., 2013
Transfection with ChFP-HTTExon1Q25 and exposure to HTTExon1Q44 fibrils	Undifferentiated and differentiated N2A	Seeding capacity of transfected HTTExon1Q44 fibrils with ChFP-HTTExon1Q25 in undifferentiated and differentiated N2A cells	Ruiz-Arlandis et al., 2016
PolyQ (KKQ30KK or KKQ40KK) oligomers	HTT14A2.6	Seeding of polyQ oligomers in HTT14A2.6 cells	Tan et al., 2015
Exposure to media and lysates from induced HTT14A2.6 cells	Naïve	Media and lysates from induced HTT14A2.6 cells can seed aggregation in naïve cells	
CSF from deceased HD patients	HTT14A2.6	CSF obtained from HD patients postmortem increase aggregate number in HTT14A2.6 cells	
CSF from BACHD rats	HTT14A2.6	CSF from living BACHD rats can seed aggregation	
Expression of PrDQ19, PrDQ54 and PrDQ92	GT17	Soluble Sup35 protein converts into insoluble aggregates following expression of PrDpolyQ pathogenic (≥54 glutamines) proteins	Goehler et al., 2010
Transfection with Rnq1Q19, Rnq1Q54 and Rnq1Q91	GT17	Pathogenic PolyQ tracts convert soluble Rnq1 into insoluble aggregates	
Transfection with HTT25Q-/103Q-GFP	74-D694	HTT103Q induce insoluble aggregates of Def1, Pub1, Rpn10, Bmh2, Sgt2, and Sup35 proteins	Nizhnikov et al., 2014
Transfection with HTT25Q-/103Q-GFP	BY4742 and 74-D694	HTTQ103 promotes it own aggregation and that of Sup35 and Def1 in different yeast strains Deletion of Def1, which normally enhances mHTT aggregation and toxicity, decreases selectively the amount of polymerized HTTQ103 and its cytotoxic effect in BY4742 cells	Serpionov et al., 2017

*BACHD, bacterial artificial chromosome (BAC) transgenic rat model of HD; Bmh2, protein BMH2; BY4742, yeast strain**;** C17.2 cells, murine C17.2 neural precursor cells; c-Myc, c-Myc tag peptide; CFP, cyan fluorescent protein; ChFP, mCherry fluorescent protein; Cos-1, fibroblast-like cell lines derived from monkey kidney; Cryo-ET, cryo-electron tomography; Cryo-FLM, cryogenic-fluorescent light microscopy; CSF, cerebrospinal fluid; Def1, RNA polymerase II degradation factor 1; EGFP, enhanced green fluorescent protein; FITC, fluorescein isothiocyanate; GFP, green fluorescent protein; GT17, yeast strain; HA-tag, human influenza hemaglutinin tag; HD, Huntington's disease; HEK, human embryonic kidney cells; HeLa, human uterine cervical carcinoma cells; HTT14A2.6:PC12 cells, (cell line from pheomochromocytoma) that inducibly express a fragment of mHTT (truncated exon 1) epitope tagged with enhanced green fluorescent protein (mHTTex1-GFP) in the presence of ponasterone A; N2A, murine neuroblastoma cell line; PolyQ, polyglutamine; PrD, prion domain; Pub1, nuclear and cytoplasmic polyadenylated RNA-binding protein; Rnq1, yeast prion protein; Rpn10, proteasome regulatory particle base subunit RPN10; Sgt2, small glutamine-rich tetratricopeptide repeat-containing protein 2; Sup35, yeast eukaryotic release factor 3; YFP, yellow fluorescent protein*.

## mHTT propagation takes place by unknown mechanisms

The seeding process of synthetic mHTT fibrils has been studied in greater detail with bimolecular fluorescence complementation assays (BiFC) alongside time-lapse microscopy, allowing for the visualization of HTTExon1 oligomer formation using halves of Venus fluorescent proteins (Herrera et al., 2011). With this approach, the reconstruction of a functional fluorophore testifies to the dimerization of mHTT fragments. A strong fluorescence signal was detected in Q103HTT-Venus transfected human glioma cells (H4), while much lower signals were measured in H4 cells expressing Q25HTT-Venus. Dimers of 103QHTT-Venus constructs appeared 30–45 min after exposure, and some cells began to show larger aggregates after only 1 h. Analyses at later time points, however, showed that all cells eventually died, indicating that both oligomers and inclusion bodies induced irreversible toxic effects. The cell-to-cell transmission potential of pathological mHTT exon1 fragment was further tested in H4 or HEK cells transfected with 103QHTT-V1 or 103QHTT-V2 plasmids and co-cultured. After 3 days, diffuse fluorescence revealed trafficking and, once again, the cell-to-cell transmission capacity of 103Q (Herrera et al., 2011; Table 1). One important point to take into consideration is that the mHTT propagation does not always require cell-to-cell contact. For example, the media of HEK cells overexpressing GFP-mHTT-Q19 or GFP-mHTT-Q103 triggers spreading of non-pathological (Q19) and pathological length (Q103) polyQ in SH-SY5Y cells after 5 days of incubation (Jeon et al., 2016; Table 2). This may indicate that, in *in vivo* contexts, mHTT could spread over extended distances and thereby exercise a toxic effect on remote cells.

Tau, α-synuclein and HTTQ50 fibrils can all be taken up by C17.2 cells and they are able to seed aggregation of intracellular tau RD-CFP/YFP, α-synuclein-CFP/YFP and HTT(Q25)CFP/YFP respectively (Table 2). In particular, internalization of tau and α-synuclein fibrils has been shown to be mediated by heparan sulfate proteoglycans (HSPGs)-binding, which stimulates cell uptake via macropinocytosis. This internalization can be inhibited by heparin, chlorate and heparinase III (Holmes et al., 2013). It should be noted, however, that such uptake has not been reported for HTTQ50 fibrils, which suggests it may use a different pathway (Holmes et al., 2013). Similarities and differences in uptake mechanisms between proteins will have to be carefully taken into account when designing treatment approaches.

## Targeting mHTT propagation mechanisms as a treatment option

Taken together, there is now compelling evidence, with a large part emerging from *in vitro* studies, that mHTT, as for other proteins linked to neurodegenerative diseases, may behave in a prion-like fashion. However, given that HD is driven by a single gene that leads to the expression of mHTT in every cell of the body, is this of any relevance to the disease burden/onset? And can these propagation mechanisms be used for therapeutic benefits?

In many disorders, including HD, transynaptic/transneuronal spreading may be the favored route of protein transfer. Exocytosis, which occurs at the synaptic terminal, is regulated by a number of proteins, some of which interact pre- or post-synaptically with HTT. Consequently, mHTT can interfere with normal synaptic transmission by sequestering the wild-type protein, a phenomenon that is exacerbated as the polyQ length increases (Smith et al., 2005). The work of Pecho-Vrieseling and colleagues has provided evidence that mHTT itself can circulate between the pre- and post-synaptic membrane. Administration of BoNT—which works by cleaving and inactivating SNARE proteins (SNAP25 and VAMP-2)—blocked exocytosis and consequently mHTT transynaptic propagation (Pecho-Vrieseling et al., 2014).

mTOR inhibitors have also demonstrated to successfully rescue cortico-striatal degeneration in organotypic-striatal cultures derived from R6/2 and Hdh^(CAG)150^ HD mouse models. mTOR regulates a wide variety of cell functions—including autophagy downregulation—and the mTOR inhibitor AZD8055 has been shown to decrease the size of mHTT aggregates and the amount of insoluble mHTT in medium-spiny neurons in both the models cited above (Proenca et al., 2013). mTOR inhibitiors have also been found to reduce mHTT accumulation and alleviate toxicity in fly and mouse models of HD (Ravikumar et al., 2004), prevent levo-dopa induced dyskinesias in mouse models of PD (Santini et al., 2009) and ameliorate cognitive deficits in various AD animal models (Wang C. et al., 2014).

Transneuronal propagation of pathological proteins is not a unique characteristic of mHTT. It has been demonstrated for tau and β-amyloid in AD (de Calignon et al., 2012; Lee et al., 2012; Wu et al., 2013) and α-synuclein in PD (Desplats et al., 2009; Angot et al., 2012; Freundt et al., 2012; Luk et al., 2012a,b; Rey et al., 2016). Of great relevance is the fact that trans-neuronal spread of α-synuclein can be blocked by the monoclonal antibody 1H7 *in vitro* (Games et al., 2014) and *in vivo* (Spencer et al., 2017), ameliorating axonal transport and synaptic trafficking, raising the possibility of also treating HD through passive immunization. While the release of vesicles containing tau, α-synuclein and TDP-43 is increased by overexpression of the co-chaperone DnaJC5—possibly through non-canonical SNAP23 exocytosis—this is not the case for mHTT, indicating that not all pathological proteins may behave in the same manner under the same circumstances (Fontaine et al., 2016). We therefore may have to tailor therapies to specifically address the different mechanisms for propagation adopted by each protein, while other treatment options may be applicable to a range of proteins.

Previous reports have also indicated that tunneling nanotubes represent an efficient means for cells to communicate and share material. As we described above, F-actin is the principal component of the nanotube trafficking paths and its depolymerization by latrunculin or by cytochalasin B (Bukoreshtliev et al., 2009) abolishes the formation of TNTs, or at least reduces the number formed. If depolymerizing actin may be an attractive target by which to prevent bridge formation between cells—hence mHTT spreading—its implications for microfilaments and microtubules within the cytoskeleton organization would need to be known before applying such methodologies, given the detrimental consequences this may have on the cell integrity.

TNT formation is also promoted by oxidative stress, as shown with H_2_O_2_ administration and serum starvation (Wang et al., 2011). However, interventions designed to control oxidative stress are too non-specific to allow for the generation of a meaningful agent. Additionally, modulations of the M-Sec promoter protein (Hase et al., 2009), tumor suppressor p53, epidermal growth factor receptor (EGFR) and EGFR-regulated Akt, PI3K and mTOR (Wang et al., 2011) could be considered as targets given they all have a direct effect on nanotube growth. Unfortunately, reduction of physiological levels of any of these transcription factors could have devastating effects on the cell's vital functions.

If aiming to block the synthesis of F-actin, oxidative stress or various proteins involved in TNT formation is not a viable approach, the motor molecules that can carry mHTT could be a target option. For example, it has been reported that the transfer of endosome-related organelles is actomyosin dependent (Gurke et al., 2008) and therefore molecular motor myosin-X (Myo10) expression increases the number of TNTs and the transfer of vesicles between co-cultured cells (Gousset et al., 2013). In particular, a specific sequence of Myo10 is required for the formation and function of TNTs (Gousset et al., 2013), and its deletion does not affect filopodia involved in cell-to-cell communication (Bohil et al., 2006). However, it is important to note that TNT formation is beginning to be understood as a process that is cell-specific, in other words different cell lines can induce TNTs via different mechanisms (Gousset et al., 2013). This is a characteristic that is extremely important to take into consideration if we aim to inhibit TNT production in a specific cell population.

Endocytosis is also a very efficient pathway for mHTT internalization (Ruiz-Arlandis et al., 2016) and therefore could be considered as a process to target to halt propagation and disease dissemination. For example, chlorpromazine, monodansylcadaverine and dynasore can suppress mHTT uptake in N2A cells by a clathrin-dependent endocytosis mechanism (Ruiz-Arlandis et al., 2016). Although chlorpromazine has been used to treat psychotic disorders since the fifties, it is known to induce side-effects which include tardive dyskinesia, dystonia, motor restlessness and akathisia, which limits its use in a debilitating movement-disorder such as HD. Other pharmacological approaches could be employed to specifically inhibit clathrin-dependent endocytosis but a complete screening should be undertaken to identify such compounds, test their efficacy and monitor their potential side-effects as blocking this pathway could affect the well-being of the cells/neurons and only partially prevent mHTT propagation.

However, blocking mHTT uptake without targeting its release would not provide a fully efficient therapy and in order to address this issue, neutral sphingomyelinase and PI3-kinase inhibitors have been investigated and shown to be capable of reducing the secretion of mHTT–more specifically of 72Q and 25Q in transfected N2A cells, full length mHTT expressing lentiviruses were transduced in rat primary cortical neurons and endogenous mHTT of HdhQ111/Hdh^+^ and HdhQ111/HdhQ111 murine striatal cells. This event occurred simultaneously with the depletion of intracellular mHTT sequestered in vesicles, highlighting, once again, the role of late endosomes/lysosomes activity in mHTT secretion. Surprisingly, the knockdown of mHTT secretion by neutral sphingomyelinase inhibitors did not increase the amount of mHTT in the intracellular compartment, neither did it induce cell toxicity, raising the possibility to target release and propagation of free forms of mHTT. The side-effects of PI3-kinase inhibitors were not reported (Trajkovic et al., 2017). The absence of intracellular mHTT accumulation may indicate the existence of other release mechanisms (Jeon et al., 2016). Indeed, mHTT is very often detected outside the cell boundary, notably in the cerebrospinal fluid (Tan et al., 2015; Wild et al., 2015) within the extracellular matrix (Cicchetti et al., 2014), in blood vessels (Drouin-Ouellet et al., 2015) and possibly in plasma (Jeon et al., 2016). mHTT found in the extracellular milieu is also likely to emerge from cell death driven by mHTT toxicity (Cicchetti et al., 2014).

The plasma membrane constitutes a major barrier to external toxins. To preserve the homeostasis of its internal compartments, it selectivity permits compounds to cross or bind to the surface. Interactions between proteins and cell membranes can be physical, as determined by electrostatic attractions, or chemical, through specific or non-specific lipid, protein or carbohydrate bindings. In particular, binding of the HTT-N-terminus with the lipids of biological membrane has been shown to dependent on electrostatic interactions (Kegel et al., 2005). mHTT carries variable repetitions of polyglutamine residues at the N-terminus, and increasing the number of polyQ offers more insertion sites into the lipid bilayer. This has been demonstrated to occur via vesicles that simulate the biological membrane, corrupting its integrity (Kegel et al., 2009). Several other studies have demonstrated plasma membrane structural modifications following interactions with tau, Aβ or α-synuclein (Zhu et al., 2003; Flach et al., 2012). These interactions are well described in studies which compared the properties of α-synuclein and mHTT fibrils. They have revealed that soluble α-synuclein fibrils have differing binding capacities depending on their charge, while HTTExon1Q41 does not express this specificity, at least as demonstrated in synthetic vesicles (Pieri et al., 2012). It was also shown that HTTExon1 binding is dependent on the number of interaction sites, which is not the case for α-synuclein (Monsellier et al., 2016). Despite some dissimilarities, α-synuclein and mHTT fibrils do share common properties such as being able to modify the permeability of the lipid membrane by Ca^2+^ influxes (Monsellier et al., 2016). Furthermore, cholesterol concentration in the phospholipid bilayer impacts on its rigidity and stability, challenging the fibrils' capacity to alter the membrane's permeability (Pieri et al., 2012). In this regard, polyunsaturated fatty acid ethyl-eicosapentaenoic acid (ethyl-EPA) available in specific foods can dampen motor deficits in a HD mouse model (Clifford et al., 2002) probably due to its capacity to positively influence the plasma membrane properties, by preventing oxidative stress (Puri et al., 2005) or inhibiting apoptotic pathways (Murck and Manku, 2007). As its metabolite docosahexaenoic acid (DHA) (Wu et al., 2008), EPA was shown to modulate synaptic plasticity in *ex vivo* hippocampal slices from rats following the administration of ethyl-EPA for a period of 8 weeks (Kawashima et al., 2010). This compound has already completed a phase III clinical trial with some evidence of efficacy as shown by changes in the total motor scores in human patients with >45 CAG repeats (Puri et al., 2005).

## Perspectives

In this review, we have presented the main findings from *in vitro* models, with a particular emphasis on mHTT propagation and seeding capacity, as it is becoming increasingly apparent that these mechanisms play a significant role in disease onset and manifestation and that targeting them may offer viable treatment options—keeping in mind that additional *in vivo* work must be conducted to go forward with the development of procedures that target this aspect of the disease. In the last few years, the focus of novel therapeutic approaches has been shifted and the aim is now to decrease the amounts of mHTT using approaches such as gene silencing (Lu and Yang, 2012) and editing (Yang et al., 2017). However, considerable challenges remain which relate to the capacity of mHTT to propagate between cells. Approaches designed to block propagation would not interfere with the primary aim of gene silencing/editing methodologies. This is particularly true in light of the fact that abnormalities in HD also lie outside the CNS (Sassone et al., 2009; Carroll et al., 2015). Future therapeutic perspectives will need to converge and tackle this genetic disorder from several fronts, including both pre-manifest and manifest patients, using combinations of agents that inhibit mHTT synthesis; restrict the damaging effects of the protein portion that is then secreted and by so doing prevent the accumulation and spread of this pathogenic protein.

## Author contributions

MM was responsible for conducting the literature search as well as designing tables and figures. FC conceptualized and wrote the manuscript.

### Conflict of interest statement

The authors declare that the research was conducted in the absence of any commercial or financial relationships that could be construed as a potential conflict of interest.
